# The Anatomy of the Urinary Bladder and Perinæum of the Male

**Published:** 1842-10

**Authors:** 


ART. XVII.—*The Anatomy of the Urinary Bladder and Perinæum of the Male.* Illustrated by Engravings. *With Physiological, Pathological, and Surgical Observations.* By ALEXANDER MONRO, M.D. F.R.S.E. &c., Professor of Anatomy in the University of Edinburgh, &c. —*Edinburgh,* 1842. 8vo, pp. 90.

WE have read with becoming care a great part of the above work, and though we willingly admit that the Professor has redeemed the pledge put forth in his title-page, and that we have found but little to criticise, yet we have risen from our task with the old query of—*cui bono?* Was the volume (if such we may term it) which we have been perusing wanted? Has it filled a vacuum or superseded other similar works? However, these are questions which only a small number of authors can answer satisfactorily in the affirmative—it is a test too searching for a vast proportion of the books which issue from our modern press, and therefore one to which we would not desire to bring the production before us; it must stand or fall, as others do, on its own individual merits.

The plan and object of Dr. Monro's treatise are similar to those of Mr. Morton's work, which we had occasion to notice in a preceding Number of our Journal, (Vol. VIII. p. 244.) The anatomical descriptions are sufficiently long without being tedious, and the practical remarks on the various operations and their results are for the most part good, without however, we should say, possessing the peculiar character which familiarity with practice is alone enabled to impart. Indeed in some few examples this fault might mislead students: for instance, in the section devoted to retention of urine and the operation of puncturing the bladder, no notice is taken of the frequent consequence of obstinate stricture, viz. extravasation of urine, or of the ordinary method now adopted for its relief by division of the stricture in the perineum, an operation which possesses the double advantage of being palliative and curative, and yields in importance to no one operation on the urinary organs; but these are faults of omission.

The descriptions are in many parts graphic, and in some instances somewhat antiquated, as is also the matter. The references are numerous, and many of them to authors well-nigh forgotten in modern surgery. The woodcuts do not rise above mediocrity. Yet on the whole, we do not deny that the student and young practitioner may meet with many valuable hints in Dr. Monro's treatise; and certainly with many quoted opinions which would occupy much of his time to collect for himself.

